# Dapagliflozin, Liraglutide, and Their Combination Attenuate Diabetes Mellitus-Associated Hepato-Renal Injury—Insight into Oxidative Injury/Inflammation/Apoptosis Modulation

**DOI:** 10.3390/life12050764

**Published:** 2022-05-21

**Authors:** Mohamed El-Sherbiny, Mohamed El-Shafey, Eman Said, Gehan Ahmed Shaker, Mohamed El-Dosoky, Hasnaa Ali Ebrahim, Sally Yussef Abed, Khalid M. Ibraheem, Ahmed Mohsen Faheem, Muntazar AlMutawa, Bayader Alatawi, Nehal M. Elsherbiny

**Affiliations:** 1 Department of Basic Medical Sciences, College of Medicine, AlMaarefa University, P.O. Box 71666, Riyadh 11597, Saudi Arabia; msharbini@mcst.edu.sa (M.E.-S.); 191120392@student.mcst.edu.sa (M.A.); 2 Department of Anatomy and Embryology, Faculty of Medicine, Mansoura University, Mansoura 35516, Egypt; mmelshafey@fcms.edu.sa; 3 Physiological Sciences Department, Fakeeh College for Medical Sciences, Jeddah 21461, Saudi Arabia; 4 Department of Pharmacology and Toxicology, Faculty of Pharmacy, Mansoura University, Mansoura 35516, Egypt; emansaid@mans.edu.eg; 5 Faculty of Pharmacy, New Mansoura University, New Mansoura 7723730, Egypt; 6 Department of Medical Physiology, Faculty of Medicine, Mansoura University, Mansoura 35516, Egypt; gehanshaker@mans.edu.eg; 7 Department of Neuroscience Technology, College of Applied Medical Science in Jubail, Imam Abdulrahman Bin Faisal University, Jubail 34221, Saudi Arabia; mesalama@iau.edu.sa; 8 Department of Basic Medical Sciences, College of Medicine, Princess Nourah bint Abdulrahman University, P.O. Box 84428, Riyadh 11671, Saudi Arabia; haebrahim@pnu.edu.sa; 9 Department of Respiratory Care, College of Applied Medical Science in Jubail, Imam Abdulrahman Bin Faisal University, Jubail 35811, Saudi Arabia; syabed@iau.edu.sa; 10 Department of Anaesthesia Technology, College of Applied Medical Sciences in Jubail, Imam Abdulrahman Bin Faisal University, Jubail 35811, Saudi Arabia; kmibraheem@iau.edu.sa; 11 Department of Medical Biochemistry and Molecular Biology, Faculty of Medicine, Mansoura University, Mansoura 35516, Egypt; amfaheem@fcms.edu.sa; 12 PharmD Program, Faculty of Pharmacy, University of Tabuk, Tabuk 71491, Saudi Arabia; 381007679@stu.ut.edu.sa; 13 Department of Pharmaceutical Chemistry, Faculty of Pharmacy, University of Tabuk, Tabuk 71491, Saudi Arabia; 14 Department of Biochemistry, Faculty of Pharmacy, Mansoura University, Mansoura 35516, Egypt

**Keywords:** dapagliflozin, liraglutide, NF-κB, hepato-renal, cleaved caspase-3

## Abstract

In this study, we aim to explore the beneficial therapeutic impacts of dapagliflozin (Dapa), a highly potent, reversible, and selective sodium–glucose cotransporter-2 inhibitor, and liraglutide (Lira), a glucagon-like peptide-1 (GLP-1) receptor agonist, as hypoglycaemic agents for the management of diabetes mellitus (DM), as well as their combination against DM-induced complications, including hepato-renal injury. Indeed, the progression of DM was found to be associated with significant hepatic and renal injury, as confirmed by the elevated biochemical indices of hepatic and renal functions, as well as histopathological examination. Dapa, Lira, and their combination effectively attenuated DM-induced hepatic and renal injury, as confirmed by the recovery of hepatic and renal functional biomarkers. The administration of both drugs significantly reduced the tissue contents of MDA and restored the contents of GSH and catalase activity. Moreover, NF-κB and TNF-α expression at the protein and gene levels was significantly reduced in the liver and the kidney. This was in parallel with the significant reduction in the caspase-3 content in the liver and the kidney, as well as suppressed cleaved caspase-3 expression in the hepatic and renal specimens, as confirmed by immune–histochemical analysis. Notably, the combined Dapa/Lira treatment demonstrated an additive superior hepato-renal protective impact compared with the use of either drug alone. Thus, it appears that Dapa and Lira, through the coordinated modulation of oxidative, inflammatory, and apoptotic signalling, confer a significant hepato-renal protective impact against DM-induced complications and tissue injury.

## 1. Introduction

Diabetes Mellitus (DM) is a chronic metabolic disorder characterized mainly by hyperglycaemia. It is usually accompanied by a deficiency in either insulin secretion or action. DM is considered a major global critical health care problem. According to the International Diabetes Federation, the number of diabetic patients is expected to increase to an estimated 552 million by 2030 [1]. Hyperglycaemia— the main change in diabetes— is associated with major alterations in the metabolism glucose and fat, and increased oxidative stress, that all have a role in the progression of the complications of diabetes. In addition to kidney disease, patients with diabetic nephropathy (DN) may have impaired hepatic functions [2].

Long-term hyperglycaemia promotes enhanced general oxidative status and increases in the incidence of organ dysfunction, especially diabetic nephropathy and liver diseases [3]. DM is considered to be among the most common causes of liver disease and is an important cause of death in diabetic people [4]. The enhanced oxidative status prompts the development of liver diseases through the induction of hepatocyte apoptosis, hepatic inflammatory response, and fibrogenesis [5]. Free radical generation and the suppression of antioxidant defences have been reported to be involved in the pathogenesis of liver damage in diabetic patients [6]. Indeed, hepatopathies, such as liver fibrosis, the deposition of fat and glycogen, and the induction of hepatic enzymes, have been reported in diabetes [7]. 

Moreover, DM progression has been associated with both microvascular (e.g., retinopathy, neuropathy, and nephropathy) and macrovascular complications as heart attacks, cerebrovascular accidents, and peripheral vascular disorders [8]. Diabetic nephropathy (DN) is a complication associated with microvascular changes that can lead to end-stage kidney disease. Multiple factors are involved in the onset of DN, of which oxidative stress plays a crucial role [9]. Glomerular sclerosis, renal tubular atrophy, and accumulation of renal interstitial fibers are the primary pathological alterations associated with DN [10]. Nevertheless, DM is linked with arterial hypertension, which results in progressive kidney damage and renal dysfunction [11]. Glomerular damage and proteinuria are prominent signs of DN, which can mostly be attributed to defects in the glomerular filtration barrier, renal inflammation, and apoptosis [12].

Thus, given the increased risk of hepato-renal injury and dysfunction post-DM progression, it has become necessary to investigate drugs that can attenuate the deleterious impact of DM on liver and kidney functions, particularly those used for the management of hyperglycaemia in diabetic patients. As a result, the goal of this study was to evaluate the effects of dapagliflozin (Dapa), liraglutide (Lira), and their combination on the onset of hepatic and renal complications in experimentally induced Type 1 DM.

Dapa is a very powerful, reversible, and selective sodium–glucose cotransporter-2 inhibitor, that is currently used in the management of type II DM. In numerous well-designed clinical studies, Dapa (as single therapy and when combined to other hypoglycaemic drugs) induced effective antihyperglycemic activity and decreased blood pressure. Furthermore, in patients with atherosclerotic cardiovascular diseases, it lowered the incidence of cardiovascular-related death or hospitalization for cardiac failure, as well as the risk of renal disease progression, when compared to placebo [13]. 

The other drug under investigation is liraglutide (Lira), a glucagon-like peptide-1 (GLP-1) receptor agonist that is administered by subcutaneous injection. It was approved in 2010 by the FDA as an adjunct therapy to diet and exercise for the management of type II DM. However, results from clinical trials have repeatedly confirmed the ability of GLP-1 analogues to induce weight loss [14]. The administration of Lira delays gastric emptying, decreases food intake, promotes the glucose-dependent production of insulin, and inhibits the release of glucagon helping to maintain blood glucose levels steady. Furthermore, lira has been shown to regulate serum lipid levels and decrease weight gain in obese T2DM patients.

## 2. Materials and Methods

### 2.1. Animals and Experimental Design

In this study, adult male Sprague Dawley rats weighing 180–220 gm and aged 8 weeks were used. The animals were acclimatized for one week under standard temperature (21 ± 2 °C), humidity (65 ± 5%), and lighting (12 h light and 12 h dark) conditions and allowed free access to a standard pelleted diet and clean drinking water. The rats were rendered diabetic by a single intraperitoneal (I.P.) injection of 50 mg/kg streptozotocin (STZ) (Sigma-Aldrich, St. Louis, MO, USA), which was freshly dissolved in citrate buffer (pH 4.5). Three days later, the blood glucose levels of the injected animals were checked using a commercially available glucometer (ACCU-CHECK Active Glucose Monitor, Roche, Germany), and animals presenting a blood glucose level higher than or equal to 250 mg/dL were considered diabetic and further used in the experiment. 

After the confirmation of diabetes, random allocation of the diabetic animals into four groups was done (*n* = 8 per every group): Group I (DM), in which the animals received vehicle (saline) daily for eight weeks; Group II (DM + Dapa), in which the animals received dapagliflozin orally (FORXIGA, AstraZeneca, Mississauga, ON, Canada; 1 mg/kg/day); Group III (DM + Lira), in which the animals received liraglutide subcutaneously (VICTOZA, Novo Nordisk, Bagsvaerd, Denmark; 0.4 mg/kg/day); lastly, Group IV (DM + Dapa and Lira), in which the animals received both Dapa and Lira daily for eight weeks. An additional eight healthy animals were injected with vehicle for eight weeks, serving as the normal control. The selection of doses and treatment timeline were in accordance with previous work conducted by our team. Procedures for the care and use of animals were in accordance with and approved by the research ethics committee of the Faculty of Medicine, Mansoura University, Mansour, Egypt.

Following the last treatment, the animals were moved to be housed individually in metabolic cages for 24 h urine collection. Thereafter, the animals were sacrificed under anaesthesia (an overdose of thiopental sodium, 40 mg/kg), and blood was collected (by retro-orbital puncture) and centrifuged for 15 min at 3000 rpm in order to separate the serum for further biochemical analyses. The liver and kidneys were harvested. The right kidney and a liver lobe were appropriately fixed in 10% neutral buffered formalin for histological and immunohistochemical studies. The left kidney was cut longitudinally; one half was homogenized in phosphate-buffered saline to be used for oxidative stress measurements, and the other half was flash frozen to be further used in the real-time polymerase chain reaction. The two liver lobes were processed similarly. 

### 2.2. Assessement of Liver and Kidney Function

Serum alanine amino transferase (ALT, ELITech Clinical Systems, Zone Industrielle, Sées, France), aspartate aminotransferase (AST, ELITech Clinical Systems, Zone Industrielle, Sées, France), albumin, bilirubin (BioMed Diagnostic, Badr City, Egypt), creatinine, blood urea nitrogen (BUN, Biodiagnostic, Giza, Egypt), and proteinuria (SPINREACT, Ctra.Santa Coloma, Spain) were assessed according to the manufacturer’s instructions.

### 2.3. Assessment of Oxidative Stress

Liver and kidney homogenates were used for measuring the malondialdehyde (MDA) content, as well as decreased glutathione (GSH) as well as catalase (CAT), following the manufacturer’s protocols (Biodiagnostic, Giza, Egypt). 

### 2.4. Real Time Polymerase Chain Reaction (RT-PCR)

Hepatic and renal tissue specimens were used to extract RNA using Direct-zol RNA Miniprep Plus (ZYMO RESEARCH CORP. USA, Cat# R2072). The extracted RNA was tested for quantity and quality using a Beckman dual spectrophotometer (Irvine, CA, USA). Complementary DNA (cDNA) was synthesized from RNA using a SuperScript IV One-Step RT-PCR kit (Thermo Fisher Scientific, Waltham, MA, USA, 12594100) according to the manufacturer’s protocol. Then, cDNA was amplified by a RT-PCR reaction in the presence of SensiFAST™SYBR—the designed primers are shown in Table 1. Relative quantification of the target genes was performed based on the 2^−∆∆Ct^ method, where Ct is the cycle threshold. 

### 2.5. Histological Investigation

Fixed tissue specimens were embedded in paraffin wax blocks after dehydration by graded ethanol, sectioned (5 µm thickness), mounted on glass slides, and stained with haematoxylin and eosin (H&E). Thereafter, the prepared stained sections were examined for histological changes, and images were captured using an Olympus light microscope (Olympus, Tokyo, Japan).

### 2.6. Immunohistochemistry

Next, 4 µm slices were prepared from the paraffin-embedded tissue specimens. The sections were deparaffinised, rehydrated in graded ethanol, and then washed with phosphate-buffered saline (PBS). Thereafter, the deparaffinized slides were microwave heated in the presence of citrate buffer solution for antigen retrieval. Then, endogenous peroxidase activity was blocked by incubation for 10 min with 3% H_2_O_2_. Sections were then blocked with 3% bovine serum albumin in PBS. The immunoreaction was induced by overnight incubation with antibody for inflammatory markers NF-κB/p56 (Thermo Fisher Scientific Inc., Waltham, MA, USA, PA5-16545), TNF-α (Santa Cruz, CA, USA, sc-52746), and apoptotic marker cleaved caspase-3 (Wuhan Servicebio Biotechnology, Wuhan, China, GB11532) at 4 °C. This was followed by incubation with the corresponding secondary antibody for 30 min. After washing, sections were stained with a 3,3-diaminobenzidine solution for colour development and counterstained with haematoxylin. Images were examined and captured using an Olympus light microscope (Olympus, Tokyo, Japan).

### 2.7. Statistical Analyses

Statistical analysis was carried out using GraphPad prism software (GraphPad Inc., San Diego, CA, USA). The results are expressed as mean ± SEM (standard error of mean). ANOVA (The analysis of variances) test was used to find statistical differences between experimental groups, followed by Tukey’s post-hoc analysis. A *p*-value of less than 0.05 was considered statistically significant. 

## 3. Results 

### 3.1. Dapa and/or Lira Treatment Improved Hepatic and Renal Function in Diabetic Rats

**Figure 1:**
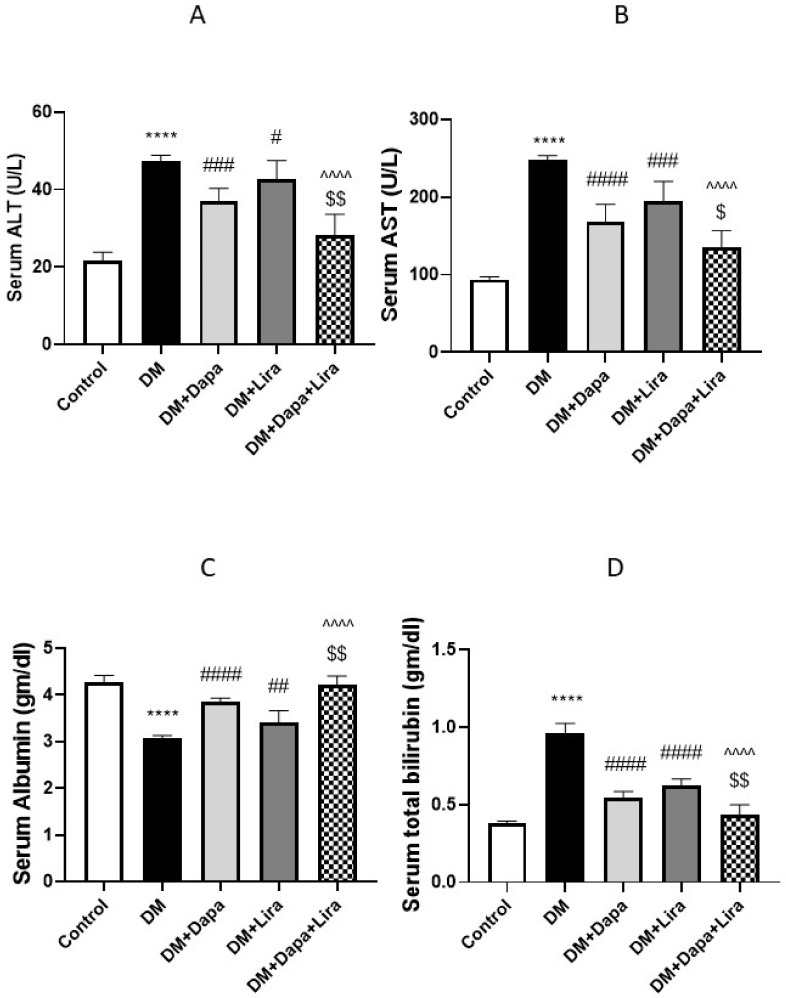
Modulatory effect of Dapa and/or Lira administration on liver function in diabetic rats after eight weeks: (**A**) serum alanine aminotransferase (ALT); (**B**) serum aspartate aminotransferase (AST); (**C**) serum albumin; and (**D**) serum total bilirubin. Data are expressed as the mean ± SE. **** represents significance when compared with the normal control (*p* < 0.0001); # represents significance when compared with the diabetic group (*p* < 0.05), ## (*p* < 0.01), ### (*p* < 0.001) and #### (*p* < 0.0001); $ represents significance when compared with the Dapa group (*p* < 0.05) and $$ (*p* < 0.01); and ^^^^ represents significance when compared with the Lira group (*p* < 0.0001).

**Figure 2:**
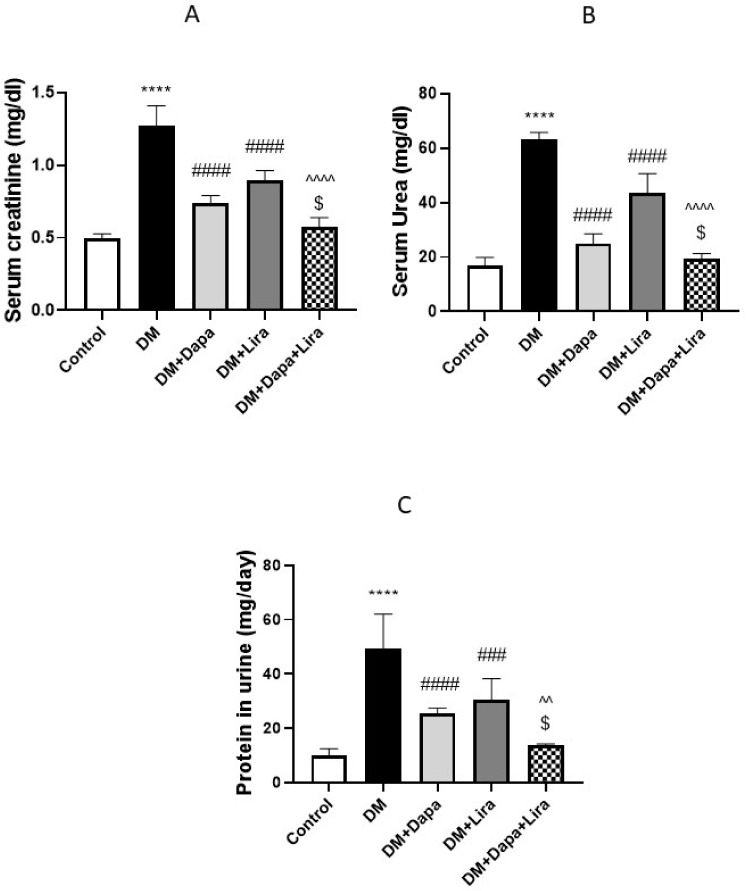
Modulatory effect of Dapa and/or Lira injection on kidney function in diabetic rats after eight weeks: (**A**) serum creatinine; (**B**) serum blood urea nitrogen (BUN); and (**C**) proteinuria. Data are expressed as the mean ± SE. **** represents significance compared with the normal control (*p* < 0.0001); ### represents significance when compared with the diabetic group (*p* < 0.001) and #### (*p* < 0.0001); $ represents significance when compared with the Dapa group (*p* < 0.05); and ^^ represents significance when compared with the Lira group (*p* < 0.01) and ^^^^ (*p* < 0.0001).

The induction of diabetes resulted in deteriorated hepatic function, as revealed by significant increases in the serum liver enzyme activities and bilirubin accompanied by a significant decrease in serum albumin (see Figure 1A–D). Concomitantly, renal dysfunction was evidenced by a significant increase in serum creatinine and BUN, accompanied by increased protein excretion in urine (see Figure 2A–C). In addition, the administration of either Dapa or Lira for 8 weeks resulted in the significant improvement of the hepatic and renal function tests, as confirmed by the estimated parameters. Additionally, the combined treatment significantly ameliorated hepatic and renal dysfunction compared with either single treatment.

### 3.2. Dapa and/or Lira Treatment Attenuated Diabetes-Induced Hepatic and Renal Cellular Injury 

**Figure 3:**
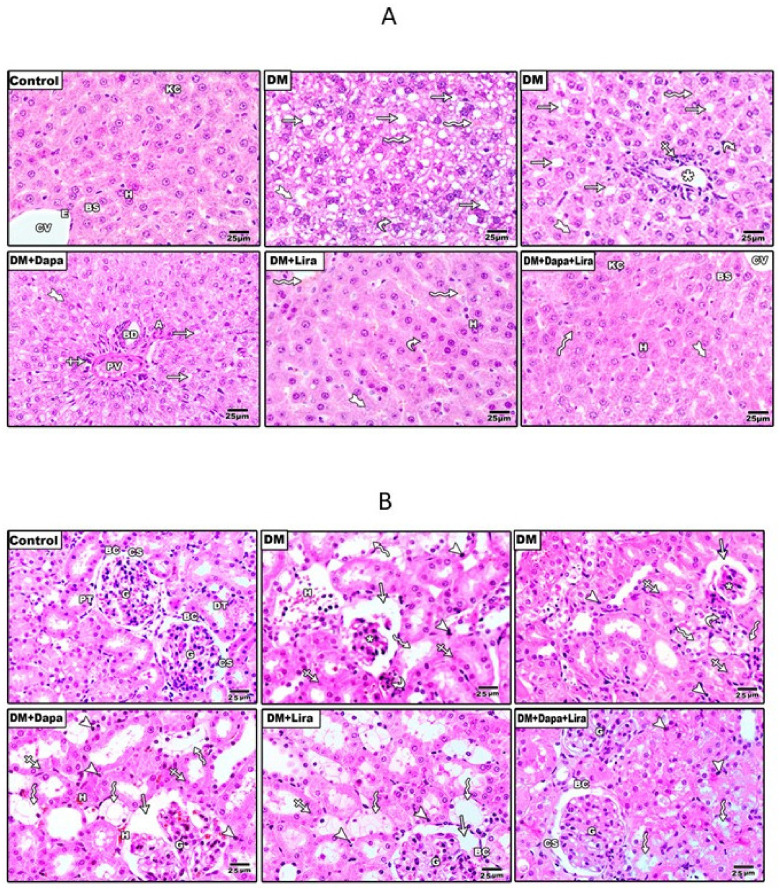
(**A**) Microscopic pictures of H&E-stained liver sections showing normally arranged hepatocytes (H) in radial plates around central veins (CV) lined with normal endothelial lining (E), normal sinusoids (BS), and Kupffer cells (KC) in the control group. The liver sections of the diabetic group (DM) show fat droplets (arrows), microvascular steatosis (curved arrows), and leukocytic cellular infiltration (crossed arrow) around a congested central vein (asterisk), haemorrhage (zigzag arrows), and the nuclei of some hepatocytes appear pale (thick arrows). The degree of injury in the treatment groups looks to be moderate in DM + Dapa, mild in DM + Lira, and more or less normal in the DM + Dapa + Lira group. X: 400; Bar = 25 µm. (**B**) Photomicrograph of H&E-stained renal tissues. CONTROL group: normal renal cortex showing renal corpuscle (G: glomerulus, CS: capsular space, and BS: Bowman’s capsule), distal tubules (DT), and proximal tubules with a brush border of intact lining cells. The renal cortex of the DM group showed shrunken glomerular capillaries (asterisk) and widening of the capsular space (arrow), as well as dilated tubules that have wide lumens and loss of their apical brush border (zigzag arrows). Haemorrhage areas are present in the lumen of some tubules (H). Pyknotic cells are also present (arrow heads). Some cells appear pale and have lost their rounded shape (crossed arrows). The treated groups showing moderate injury in DM + Dapa and mild injury in DM + Lira, while DM + Dapa + Lira appeared more or less normal, apart from some pyknotic cells. X: 400; Bar = 25 µm.

Figure 3 shows H&E-stained liver and kidney sections from different experimental groups. The inspection of the stained sections showed marked hepato-renal injury in specimens from the diabetic group, as indicated the by fat droplets, microvascular steatosis, leukocytic infiltration around the congested central vein, haemorrhage, and pale nuclei of some hepatocytes in liver specimens (see Figure 3A). Additionally, renal specimens from the diabetic group showed shrunken glomerular capillaries, the widening of the capsular space, dilated tubules with a wide lumen and loss of the apical brush border, haemorrhage area in the lumen of some tubules, and pyknotic cells (see Figure 3B); however, the Dapa- and Lira-treated groups showed mild to moderate injury in both hepatic and renal specimens, and specimens in the combination therapy-treated group appeared more or less normal.

### 3.3. Dapa and/or Lira Treatment Combated Oxidative Stress in Hepatic and Renal Tissue of Diabetic Rats

**Figure 4:**
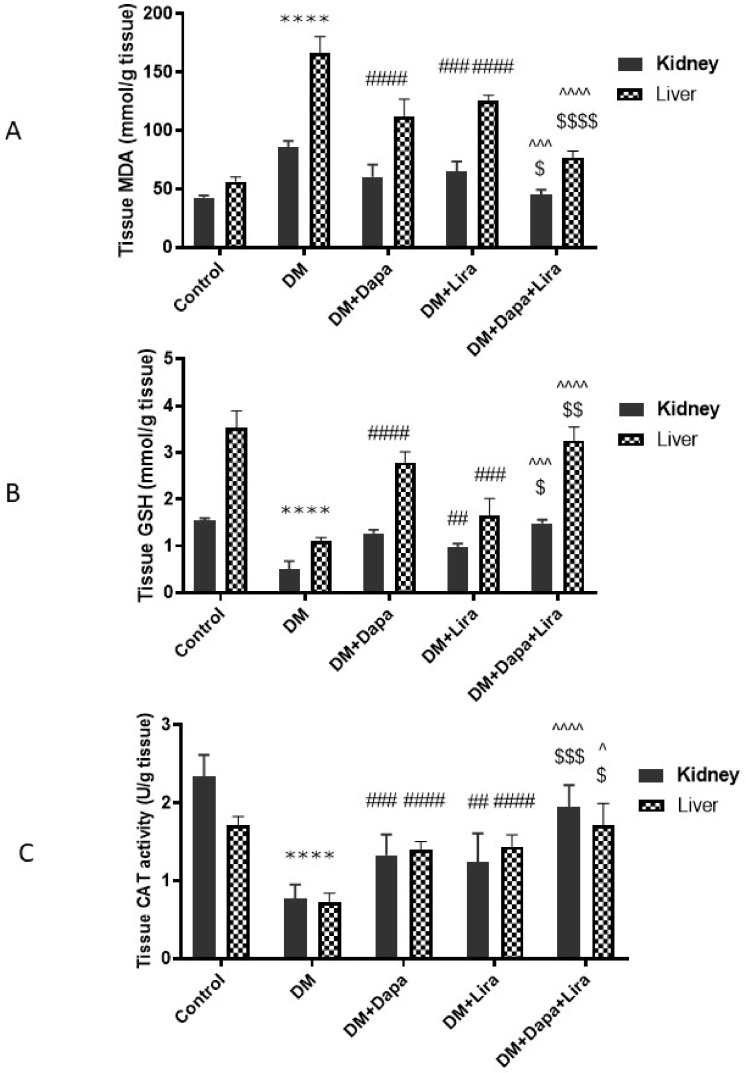
Modulatory effect of Dapa and/or Lira administration on hepatorenal oxidative stress in diabetic rats after eight weeks: (**A**) malondialdehyde (MDA); (**B**) reduced glutathione (GSH); and (**C**) catalase activity (CAT). Data are expressed as the mean ± SE. **** represents significance when compared with the normal control (*p* < 0.0001); ## represents significance when compared with the diabetic group (*p* < 0.01), ### (*p* < 0.001), and #### (*p* < 0.0001); $ represents significance when compared with the Dapa group (*p* < 0.05), $$ (*p* < 0.01), and $$$ (*p* < 0.001), $$$$ (*p* < 0.0001); and ^ represents significance in comparison with the Lira group (*p* < 0.05), ^^^ (*p* < 0.001), and ^^^^ (*p* < 0.0001).

As shown in Figure 4, diabetic rats showed marked disruption in the tissue oxidant/antioxidant balance, where hepatic and renal tissue homogenates demonstrated a significant increase in MDA (a marker of lipid peroxidation), along with marked decreases in tissue antioxidants, GSH content, and CAT enzyme activity; however, the administration of either Dapa or Lira ameliorated oxidative stress in hepatic and renal tissue, compared with the untreated diabetic rats. Moreover, the combined administration of Dapa and Lira induced more pronounced significant improvement, with an almost-normal oxidant/antioxidant balance.

### 3.4. Dapa and/or Lira Treatment Attenuated Inflammation in Hepatic and Renal Tissue of Diabetic Rats

**Figure 5:**
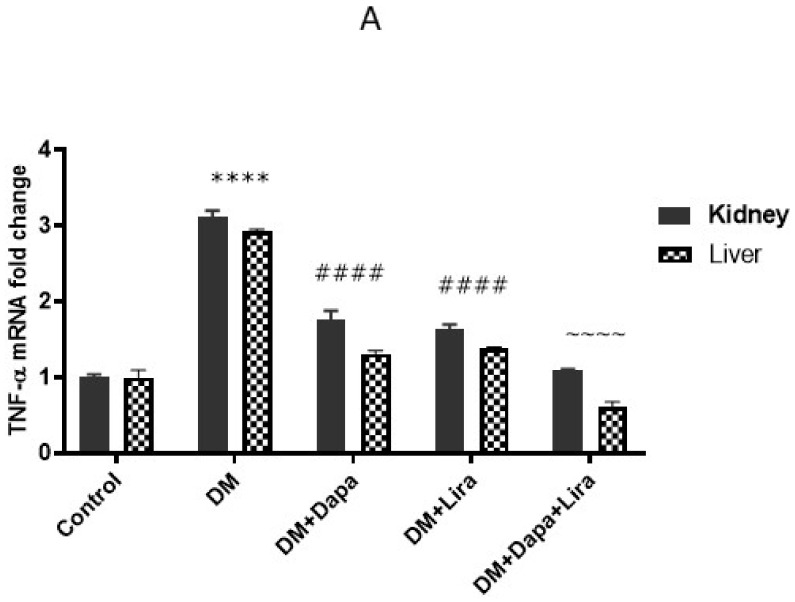

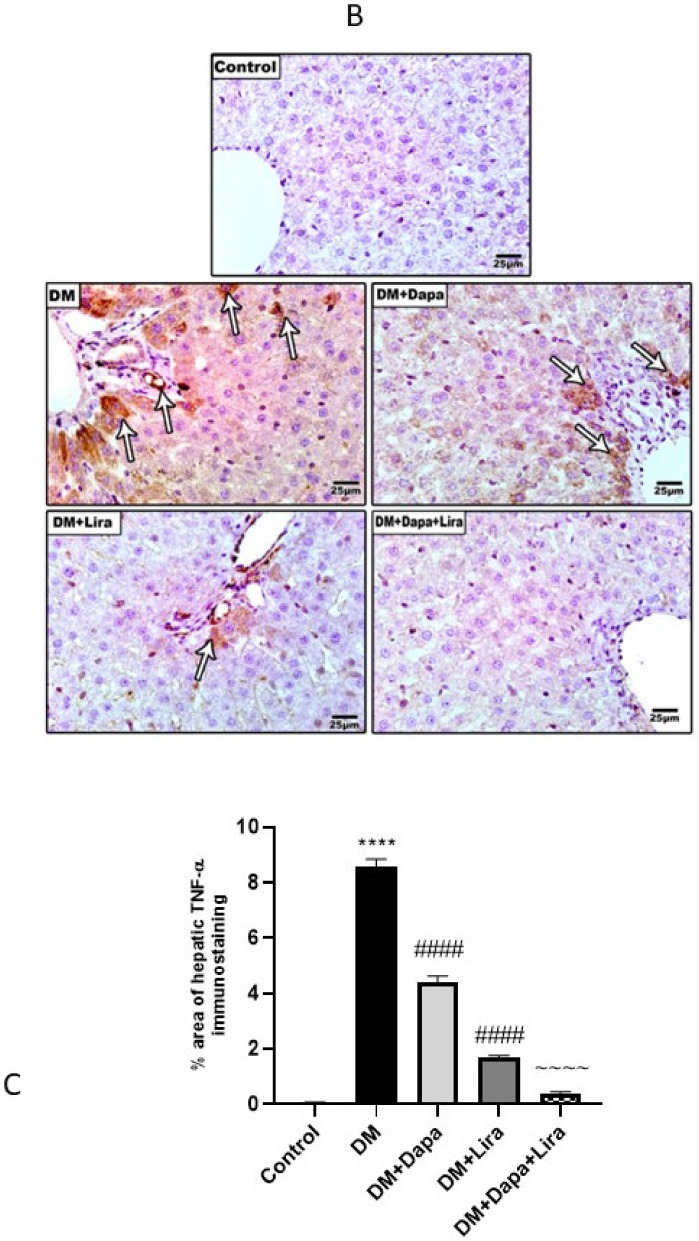

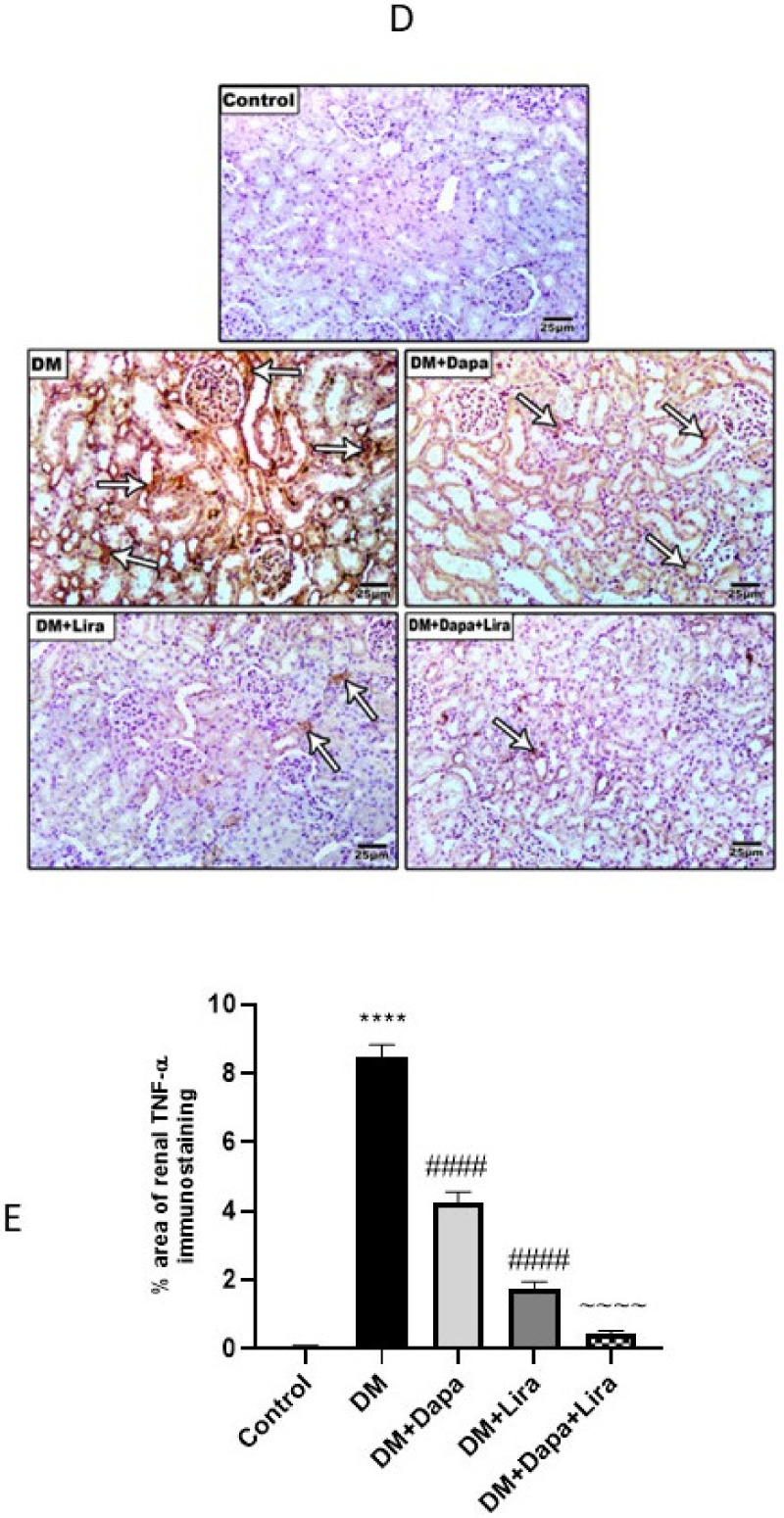
Modulatory effect of Dapa and/or Lira administration on hepatorenal inflammation in diabetic rats after eight weeks: (**A**) Tumour necrosis factor-α (TNF-α) mRNA level; (**B**) TNF-α immunostaining in hepatic specimens from different experimental groups; (**C**) % area of TNF-α immunostaining in hepatic specimens; (**D**) TNF-α immunostaining in renal specimens from different experimental groups; and (**E**) % area of TNF-α immunostaining in renal specimens. Data are expressed as mean ± SE. **** represents significance when compared with the normal control (*p* < 0.0001); #### represents significance when compared with the diabetic group (*p* < 0.0001); and ~~~~ represents significance when compared with the single-treatment groups (Dapa or Lira) (*p* < 0.0001).

**Figure 6:**
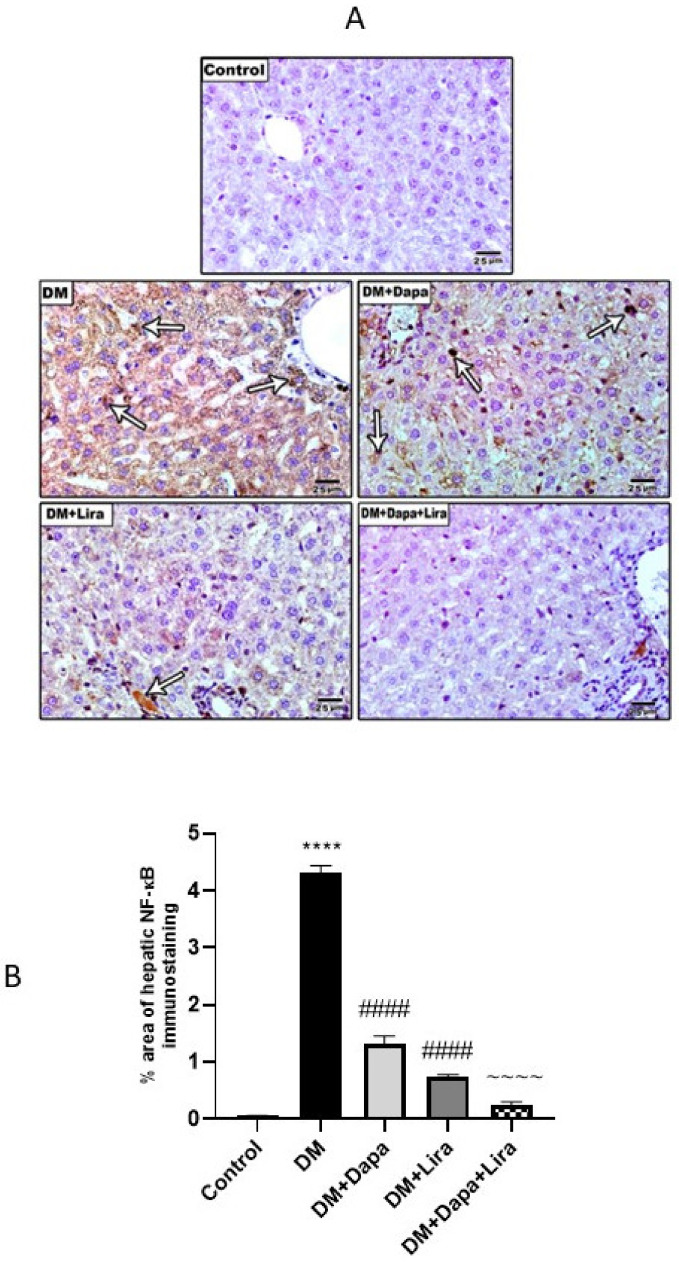

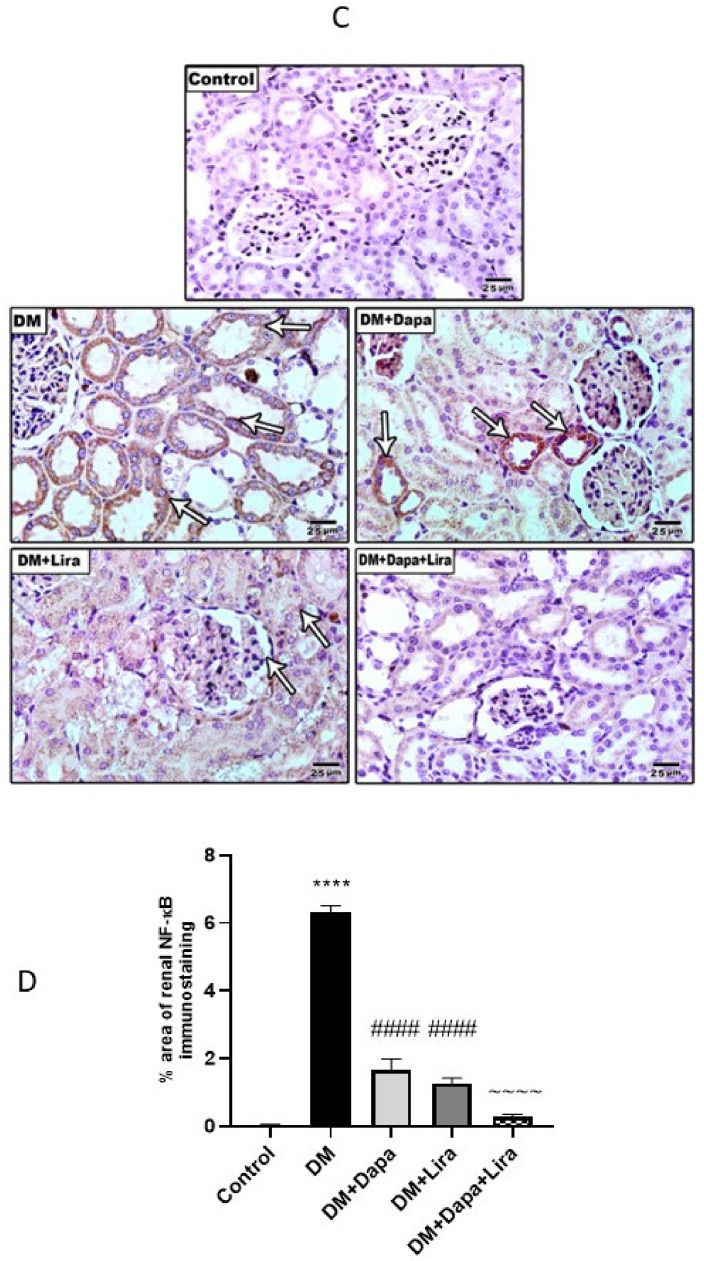
Modulatory effect of Dapa and/or Lira injection on NF-κB/p56 in diabetic rats after eight weeks: (**A**) NF-κB/p56 immunostaining in hepatic specimens from the studied groups; (**B**) % area of NF-κB/p56 immunostaining in hepatic specimens; (**C**) NF-κB/p56 immunostaining in renal specimens from the studied groups; and (**D**) % area of NF-κB/p56 immunostaining in renal specimens. Data are presented as mean ± SE. **** represents significance when compared with the normal control (*p* < 0.0001); #### represents significance when compared with the diabetic group (*p* < 0.0001); and ~~~~ represents significance when compared with the single-treatment groups (Dapa or Lira) (*p* < 0.0001).

Inflammation plays a major role in DM-induced organ injury. In line with this, the diabetic rats in the present study developed significant increases in the mRNA level and immunostaining of TNF-α, as well as a significant increase in NF-κB/p56 immunostaining in liver and kidney tissue, compared to the normal group. In contrast, this inflammatory milieu was significantly countered by either Dapa or Lira administration in diabetic animals. Furthermore, combined Dapa and Lira treatment provoked a more significant anti-inflammatory effect compared with either single treatment (see Figure 5 and Figure 6).

### 3.5. Dapa and/or Lira Treatment Attenuated Apoptosis in the Hepatic and Renal Tissue of Diabetic Rats

**Figure 7:**
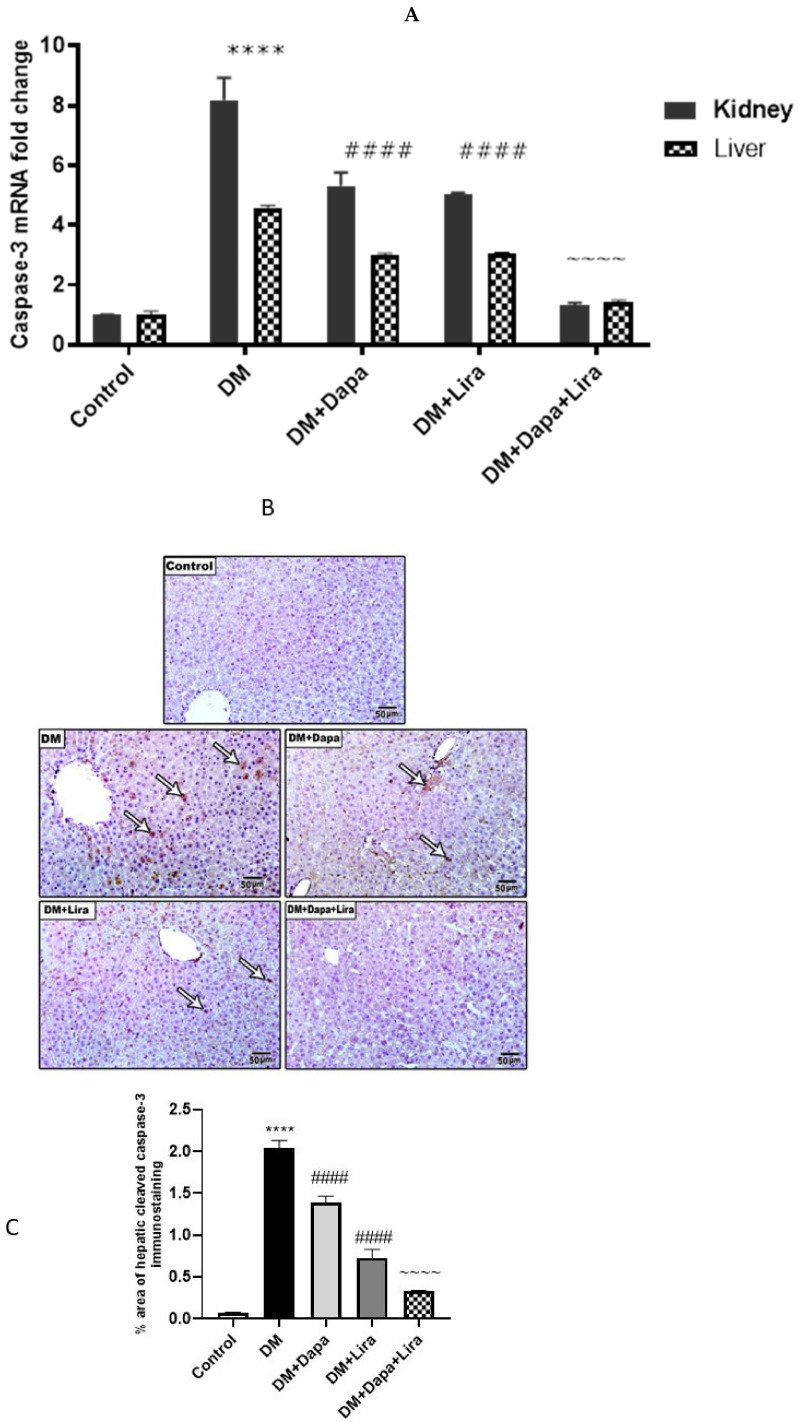

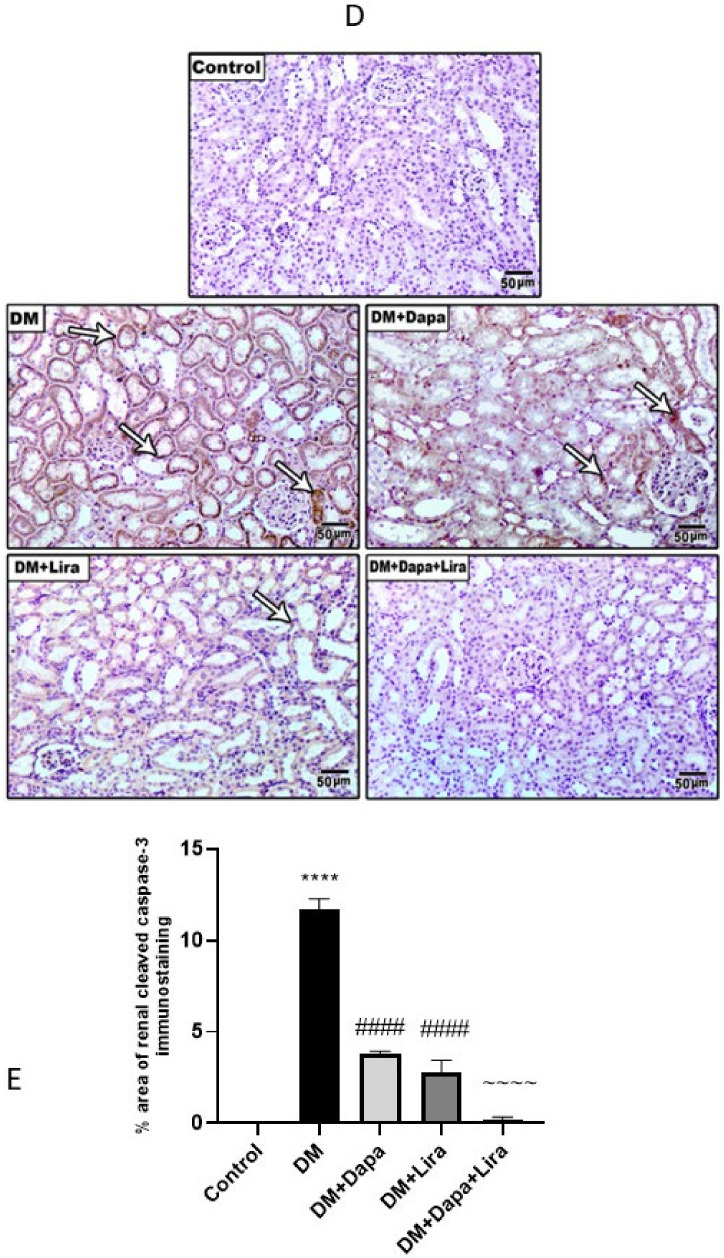
Modulatory effect of Dapa and/or Lira injection on hepatorenal apoptosis in diabetic rats after eight weeks: (**A**) caspase-3 mRNA level; (**B**) cleaved caspase-3 immunostaining in hepatic specimens from different experimental groups; (**C**) % area of caspase-3 immunostaining in hepatic specimens; (**D**) cleaved caspase-3 immunostaining in renal specimens from different experimental groups; and (**E**) % area of caspase-3 immunostaining in renal specimens. Data are presented as mean ± SE. **** represents significance when compared with the normal control (*p* < 0.0001); #### represents significance when compared with the diabetic group (*p* < 0.0001); and ~~~~ represents significance when compared with the single-treatment groups (Dapa or Lira) (*p* < 0.0001).

As indicated in Figure 7, diabetic control rats developed marked apoptosis in renal and hepatic tissues, as revealed by the significant increase in the tissue mRNA caspase-3 level and cleaved caspase-3 immunostaining when compared with the control group. In contrast, the treatment of diabetic rats with either Dapa or Lira significantly reduced the mRNA caspase-3 level and cleaved caspase-3 immunostaining in both tissues compared with the untreated diabetic control. Additionally, combined treatment with both Dapa and Lira resulted in a more significant reduction of apoptotic markers in comparison with either single treatment.

## 4. Discussion

In the current study, we highlighted the beneficial impacts of two hypoglycaemic agents—Dapa, a highly potent, reversible, and selective sodium-glucose cotransporter-2 inhibitor, and Lira, a glucagon-like peptide-1 (GLP-1) receptor agonist—as well as their combination on DM-induced renal and hepatic injuries. Either Dapa or Lira induced significant hepatic and renal recovery, as confirmed by the significant recovery of liver and renal function biomarkers with parallel histopathological recovery. Interestingly, combined Dapa/Lira treatment almost normalized the deteriorated hepatic and renal functions. 

A single I.P. injection of STZ has been well-documented to induce DM that mimics the human counterpart. The histopathological and ultra-microscopic alterations related to DN have been reported to develop at around 8 weeks [10], which the results of the current study indeed confirmed. DM-induced hepato-renal injury and the related pathologies were evident at 8 weeks post-DM induction, as confirmed through biochemical and histopathological assessments. The associations between oxidative status, inflammation, and apoptosis in DM and its associated complications have been previously demonstrated [15,16,17].

The observed ameliorative impact of either treatment was associated with parallel retractions in the oxidative tissue contents, MDA/antioxidants, GSH concentration, and CAT activity, confirming their suppressive impact on oxidative injury. Indeed, oxidative injury has repeatedly been reported to be a major contributor to DM-induced complications [18,19]. 

Indeed, Dapa treatment has been previously reported to attenuate oxidative tissue injury, which gives credence to our observations in the current study. Dapa has been shown to attenuate hypoxia/re-oxygenation-induced cardiac injury and oxidative damage through the modulation of AMPK [20], as well as attenuating cyclosporine A-induced nephrotoxicity [21]. Moreover, Dapa attenuated oxidative-injury-induced cell injury in human proximal tubular cells by reducing cytosolic and mitochondrial ROS production and altering the Ca^2+^ dynamics [22]. Moreover, Dapa has recently been reported to attenuate DM-induced cardiomyopathy, with an underlying impact on antioxidants [23].

In context, Lira has also been reported as having antioxidant properties in various experimental models. Lira ameliorated palmitate-induced oxidative injury in islet microvascular endothelial cells through the GLP-1 receptor/PKA and GTPCH1/eNOS signalling pathways [24] and ameliorated lipotoxicity-induced oxidative injury by activating the NrF2 pathway in HepG2 Cells [25]. Interestingly, Lira ameliorated erectile dysfunction, with pronounced antioxidant and ameliorative impacts on both the RhoA/ROCK pathway and autophagy in diabetic rats [26].

Noteworthy, El-Shafey et al. [23] reported an additive impact of combined Dapa and Lira treatment on DM-induced oxidative injury and DM-induced cardiomyopathy, which gives further credence to our observations in the current study.

Considering the other contributor to DM-induced injury and complications, as observed in the current study, DM progression was linked to a significant elevation in the tissue expression of the inflammatory milieu, particularly NF-κB/TNF-α. NF-κB has long been considered a prototypical pro-inflammatory signalling pathway element leading to activation by pro-inflammatory cytokines, such as TNF-α, that represents an archetypal proinflammatory cytokine which is rapidly up-regulated upon either tissue injury and/or infection. The canonical NF-κB inflammatory signalling pathway is initiated in response to either TNF-α or IL-1 signalling, which are involved in the pathogenesis of chronic inflammatory diseases [27]. Nevertheless, the cross-talk between ROS, as indicators of oxidative injury, and NF-κB signalling has been previously discussed [28].

Both Dapa and Lira treatment and their combination significantly suppressed both the hepatic and renal expression of both of NF-κB and TNF-α, confirming the suppressive impact of DM-induced canonical NF-κB activation and inflammation. It is worth noting that the suppressive effects on NF-κB and TNF-α were parallel to their suppressive impacts on oxidative injury in both organs.

The current observations are in line with those of previous studies, where Dapa was reported to attenuate TNF-α and hyperglycaemia-induced increases in intercellular adhesion molecule-1, vascular cell adhesion molecule-1, plasminogen activator inhibitor type 1, and NF-κB expression in human vascular endothelial cells [29]. Meanwhile, Lira has been reported to mitigate TNF-α-induced pro-atherogenic changes and microvesicle release in human umbilical vein endothelial cells from diabetic women [30], and exerts an anti-inflammatory action in obese patients with type 2 DM by the inhibition of the NF-kB signalling pathway [31].

Interestingly, in this study, combined Dapa/Lira treatment exerted an additive impact on the suppression of the hepatic–renal expression of the inflammatory milieu NF-κB/TNF-α; the observations of El-Shafey et al. [23] give credence to these observations.

Apoptosis is an essential mechanism that prevents prolonged inflammation [27]. The build-up of ROS is believed to be a triggering factor for the initiation of apoptosis. Meanwhile, at the cellular level, TNF-α can promote apoptosis [32]. Moreover, NF-κB has been reported to possess a pro-apoptotic role in neutrophils during inflammation [33]. Indeed, in parallel to the DM-associated increased oxidative status, and enhanced hepatic and renal expression of NF-κB and TNF-α in the DM control, the expression of caspase-3 in the liver and kidneys was increased significantly at both the genetic and the protein levels, thus confirming the incidence of apoptosis.

On the other hand, both Dapa, Lira, and their combination managed to decrease the genetic expression of caspase-3 in both the liver and kidney, suggesting their anti-apoptotic impacts. Interestingly, their combination demonstrated a superior impact compared with either treatment alone.

Indeed, Dapa has decreased the cardiac expression of cleaved caspase-3 and protected against doxorubicin-induced cardio-toxicity in breast cancer patients [34]. In pre-ischemia, Dapa conferred the maximum level of cardio-protection through decreasing cardiac apoptosis and caspase-3 expression [35], as well as attenuated experimental inflammatory bowel disease in rats and dampened colonic apoptosis by lowering the caspase-3 activity and cleaved caspase-3 expression [36]. Such observations give further credence to our observations in the current study.

Meanwhile, Lira has been shown to down-regulate caspase-3 expression and inhibit the autophagy and apoptosis induced by high glucose through GLP-1R in renal tubular epithelial cells [36], as well as to inhibit high glucose-induced oxidative stress and apoptosis in neonatal rat cardiomyocytes [37]. Moreover, Lira inhibited the increased cleaved caspase-3 in islets from diabetic rats [38]. These observations also give credence to the observed inhibitory impact of Lira on caspase-3 expression in the hepatic and renal specimens from the diabetic rats in the current study.

Thus, it appears that, through the coordinated inhibition of TNF-α mediated canonical NF-κB inflammatory signalling and the suppression of enhanced oxidative stress and apoptosis, Dapa and Lira confer significant protection against DM-induced hepato-renal injury and, as such, could be proposed as a promising therapeutic approach for diabetic patients in order to avoid long-term induced diabetic complications. In particular, the combination of both drugs exerted a significant additive hepato-renal protective impact, which contributed even more to the observed hepato-renal protective impact. 

## Figures and Tables

**Table 1:** Sequence for primers used in the present study.

Gene	Sequence	
TNF-α	Forward	5′-TAC TGA ACT TCG GGG TGA TTG GTC C-3′
	Reverse	5′-CAG CCT TCT CCC TTG AAG AGA ACC-3′
Caspase-3	Forward	5′-ATGGACAACAACGAAACCTC-3′
	Reverse	5′-TTAGTGATAAAAGTACAGTTCTT-3′
β-actin	Forward	5′-CTAAGGCCAACCGTGAAAAG-3′
	Reverse	5′-GCCTGGATGGCTACGTACA-3′

## Data Availability

Data is available on request from corresponding author.

## Sample Availability

Samples of the compounds are available from the authors.
